# MKL-GRNI: A parallel multiple kernel learning approach for supervised inference of large-scale gene regulatory networks

**DOI:** 10.7717/peerj-cs.363

**Published:** 2021-01-28

**Authors:** Nisar Wani, Khalid Raza

**Affiliations:** 1 Govt. Degree College Baramulla, Jammu & Kashmir, India; 2 Department of Computer Science, Jamia Millia Islamia, New Delhi, India

**Keywords:** Gene regulatory networks, GRN inference, large-scale GRN, Systems biology, Network biology

## Abstract

High throughput multi-omics data generation coupled with heterogeneous genomic data fusion are defining new ways to build computational inference models. These models are scalable and can support very large genome sizes with the added advantage of exploiting additional biological knowledge from the integration framework. However, the limitation with such an arrangement is the huge computational cost involved when learning from very large datasets in a sequential execution environment. To overcome this issue, we present a multiple kernel learning (MKL) based gene regulatory network (GRN) inference approach wherein multiple heterogeneous datasets are fused using MKL paradigm. We formulate the GRN learning problem as a supervised classification problem, whereby genes regulated by a specific transcription factor are separated from other non-regulated genes. A parallel execution architecture is devised to learn a large scale GRN by decomposing the initial classification problem into a number of subproblems that run as multiple processes on a multi-processor machine. We evaluate the approach in terms of increased speedup and inference potential using genomic data from *Escherichia coli*, *Saccharomyces cerevisiae* and *Homo sapiens*. The results thus obtained demonstrate that the proposed method exhibits better classification accuracy and enhanced speedup compared to other state-of-the-art methods while learning large scale GRNs from multiple and heterogeneous datasets.

## Introduction

The problem of understanding gene interactions and their influence through network inference and analysis is of great significance in systems biology (Albert, 2007). The aim of this inference process is to establish relationships between genes and construct a network topology based on the evidence provided by different data types. Among various network inference studies, gene regulatory network inference (GRNI) has remained of particular interest to researchers with extensive scientific literature generated in this domain. Gene regulatory networks (GRNs) are biological networks where genes serve as nodes and the edges connecting them serve as regulatory relations (Lee et al., 2002; Raza & Alam, 2016). Standard methods for GRN inference such as RELNET (Butte & Kohane, 1999), ARACNE (Margolin et al., 2006), CLR (Faith et al., 2007), SIRENE (Mordelet & Vert, 2008) and GENIE3 (Huynh-Thu et al., 2010) mostly use transcriptomic data for GRN inference. Among these methods, our approach is modeled along the same principle as SIRENE. SIRENE is a general method to infer unknown regulatory relationships between known transcription factors (TFs) and all the genes of an organism. It uses a vector of gene expression data and a list of known regulatory relationships between known TFs and their target genes. However, integration of this data with other genomic data types such as protein–protein interaction (PPI), methylation expression, sequence similarity and phylogenetic profiles has drastically improved GRN inference (Hecker et al., 2009). A comprehensive list of state-of-the-art data integration techniques for GRN inference has been reviewed in (Wani & Raza, 2019a).

In this article, we aim to integrate gene expression, methyl expression and TF-DNA interaction data using advanced multiple kernel learning (MKL) library provided by shogun machine learning toolbox (Sonnenburg et al., 2010) and design an algorithm to infer gene regulatory networks (GRNs). Besides, we also integrate PPI data and other data such as gene ontology information as source of prior knowledge to enhance the accuracy of network inference. The problem of network inference is modeled as a binary classification problem whereby a gene being regulated by a given TF is treated as a positive label and negative otherwise. To infer a large-scale network, the MKL model needs to be trained for each TF with a set of known regulations for the whole genome. Given *N* TFs, we need to train *N* different classification models individually and then combine the results from these models for a complete network inference task. As the number of TFs increase, the number of classification models also increase, creating resource deficiency and long execution times for the inference algorithm. The proposed approach attempts to provide a solution to this problem by distributing these classification models to different processors on a multi-processor hardware platform using parallel processing library from Python. The results from these models are stored in a shared queue object which are later on used for network inference. A detailed description of the model is contained in the methods section.

## Related Literature

An early attempt to learn and classify gene function from integrated datasets using kernel methods was carried out in Pavlidis et al. (2002). They trained a support vector machine (SVM) for gene function classification with a heterogeneous kernel derived from a combination of two different types of data (e.g., gene expression and phylogenetic profiles). Since SVM does not learn from multiple kernel matrices simultaneously, they proposed three different ways to fuse two datasets and referred to these fusion methods as (i) early integration, (ii) intermediate integration and (iii) late integration approaches. In early integration, feature vectors from heterogeneous data types are concatenated to build a single length vector for a given set of genes. This extended dataset is then transformed into a kernel matrix using appropriate kernel function and serves as an input to the SVM model from where we can draw biological inferences. In the case of intermediate integration, the two datasets are first transformed into their respective kernel matrices; subsequently these kernel matrices are added together to yield an integrated kernel for SVM training. For late integration, the authors trained the SVM models individually using the heterogeneous datasets. The probability scores which act as discriminant values obtained from separate SVM models are then added together for gene function prediction.

In fact, kernel-based methods as effective integration techniques were first proposed in Lanckriet et al. (2004), wherein a 1-norm soft margin SVM is trained for a classification problem, separating membrane proteins from ribosomal proteins. They combined heterogeneous biological datasets such as PPI, amino acid sequences and gene expression data characterizing different proteins by transforming them into multiple positive semidefinite kernel matrices using different kernel functions. Their findings reveal an improved classifier performance when all datasets are integrated as a unit compared to testing the classifier on individual datasets. In an earlier study (Lanckriet et al., 2003) on function prediction for baker’s yeast proteins, they trained an SVM classifier with multiple datasets of different types and achieved an improved performance over a classifier trained using single data type.

In yet another study for network inference using kernel data integration (Yamanishi, Vert & Kanehisa, 2004), the authors fused four different datasets, namely gene expression data, protein interaction data, protein localization data and data from phylogenetic profiles. These datasets are transformed into different kernel matrices. Datasets comprising of gene expression, protein localization and data from phylogenetic profiles were kernelized using Gaussian, polynomial and linear kernel functions. Graph datasets were kernelized using diffusion kernel (Kondor & Lafferty, 2002). This study compared both unsupervised and supervised inference methods on single and integrated datasets. To assess the accuracy of the methods, the inferred networks are compared with a gold standard protein network. Contrary to the unsupervised approaches, the supervised approach seems to make interesting predictions and capture most of the information from the gold standard. They observed that data from transcriptomic and phylogenetic profiles seem to contribute with an equal quantum of information followed by noisy PPI and localization data. Applying a supervised approach to integrated datasets seems to produce overall best results, therefore highlighting the importance of guided network inference from integrated prior biological knowledge.

In another study, Ben-Hur & Noble (2005) applied kernel methods to PPI studies and proposed a pair-wise kernel between two pairs of proteins in order to construct a similarity matrix. This pairwise kernel is based on three sequence kernels, a spectrum kernel, a motif, and a Pfam kernel. They further extended this experiment to explore the effect of adding kernels from non-sequence data, such as gene ontology annotations, homology scores and Mutual clustering coefficient (MCC) derived from protein interactions computed in each cross-validation fold. Integrating these non-sequence features with the pairwise kernel resulted in improved performance than any method by itself.

Another integration and supervised learning method that uses MKL is the Feature Selection Multiple Kernel Learning (FSMKL) proposed by Seoane et al. (2013). The feature selection is performed on variable number of features per kernel, separating feature sets from each data type with greater relevance to the given problem. The selection criteria uses statistical scoring by ranking features that are statistically aligned with the class labels and biological insights, where genes that are present in a specific pathway are chosen. They integrate gene expression, copy number variation and other genomic data from KEGG pathway. These data are transformed into their base kernels and integrated using MKL framework into a combined kernel. The prior biological knowledge in the form of pathway information serves as central criterion for FSMKL to cluster samples. The authors claim that FSMKL performance is comparable to the other state-of-the-art breast cancer prognosis methods from DREAM challenge. Speicher & Pfeifer (2015) adopted an unsupervised approach to discover cancer subtypes from an integrated kernel using MKL. The proposed method called Regularized MKL Locality Preserving Projections (rMKL-LPP) integrates multi-omics data such as gene expression, DNA methylation and miRNA expression profiles of multiple cancer types from TCGA (Tomczak, Czerwińska & Wiznerowicz, 2015). This regularized version extends the dimensionality reduction variant of the MKL technique (MKL-DR) proposed by Yan et al. (2007). The regularization term allows to use different types of kernels during optimization process and also avoids overfitting. They cluster the samples by applying k-means on the distance summation of each sample’s k-Nearest Neighbors by applying Locality Preserving Projections (LPP). Also many approaches have been proposed for parameter estimation of such large-scale and integrated models. Besides, cross validation, grid search and randomised parameter optimization methods (Remli et al., 2019) have proposed a cooperative enhanced scatter search for parameter for high dimensional biological models. Their proposed method is executed in a parallel environment and can be faster than other methods in providing accurate estimate of model parameters.

Multiple kernel Learning approach has also been applied to the domain of drug-target interaction network inference and drug bioactivity prediction. For drug-target interaction prediction, Nascimento, Prudêncio & Costa (2016) proposed a new MKL based algorithm that selects and combines kernels automatically on a bipartite drug-protein prediction problem. Their proposed method extends the Kronecker regularized least squares approach (KronRLS) (Van Laarhoven, Nabuurs & Marchiori, 2011) to fit in a MKL setting. The method uses *L*2 regularization to produce a non-sparse combination of base kernels. The proposed method can cope with large drug vs. target interaction matrices; does not require sub-sampling of the drug-target network; and is also able to combine and select relevant kernels. They performed the comparative analysis of their proposed method with top performers from single and integrative kernel approaches and demonstrated the competitiveness of KronRLS-MKL to all the evaluated scenarios. Similarly for drug bioactivity prediction (Cichonska et al., 2018) proposed pairwise MKL method in order to address the scalability issues in handling massive pairwise kernel matrices in terms of both computational complexity and memory demands of such prediction problems. The proposed method has been successfully implemented to the drug bioactivity inference problems and provides a general approach other pairwise MKL spaces.

Since MKL is applied to solve large scale learning problems, various efforts have been undertaken to devise a scheme whereby MKL algorithm can be run in a multiprocessor and distributed computational environment. The authors in Chen & Fan (2014) have proposed a parallel multiple kernel learning (PMKL) using hybrid alternating direction method multipliers (H-ADMM). The proposed method makes the local processors to co-ordinate with each other to achieve the global solution. The results of their experiments demonstrated that PMKL displays fast execution times and higher classification accuracies. Another important study to address the scalability and computational requirements in the domain of large scale learning has been carried out by Alioscha-Perez, Oveneke & Sahli (2019). They proposed SVRG-MKL an MKL solution with inherent scalability properties that can combine multiple descriptors involving millions of samples. They conducted extensive experimental validation of their proposed method on several benchmarking datasets confirming a higher accuracy and significant speedup for SVRG-MKL. In one of our recent works, we proposed a data fusion and inference model, called iMTF-GRN, based on Non-negative Matrix Tri-factorization that integrates the diverse types of biological data (Wani & Raza, 2019b). The advantage of our proposed parallel MKL-GRNI approach is that it is simple to implement and does not need complex coding to distribute multiple classification problems in a multiprocessor environment. Our method employs shared queue objects for distributing inputs and collecting outputs from multiple processors compared to PMKL (Chen & Fan, 2014) where multiple processors are explicitly made to co-ordinate using the hybrid alternating direction method of multipliers (H-ADMM) introducing complexity and an added computational overhead. Also, we chose basic addition operation to fuse multiple kernels compared to Kron-RLS MKL (Cichonska et al., 2018) method, where the fusion of multiple kernels is achieved by performing Kronecker product operation which requires calculating the inverse of individual kernels, hence a computational overhead compared to a basic arithmetic operation. Also for MKL implementation, we used the Shogun toolbox, which is a highly optimized, stable and efficient tool developed in C++ by Sonnenburg et al. (2010) making it a suitable candidate for computing-intensive and large-scale learning problems.

## Materials and Methods

**Figure 1:**
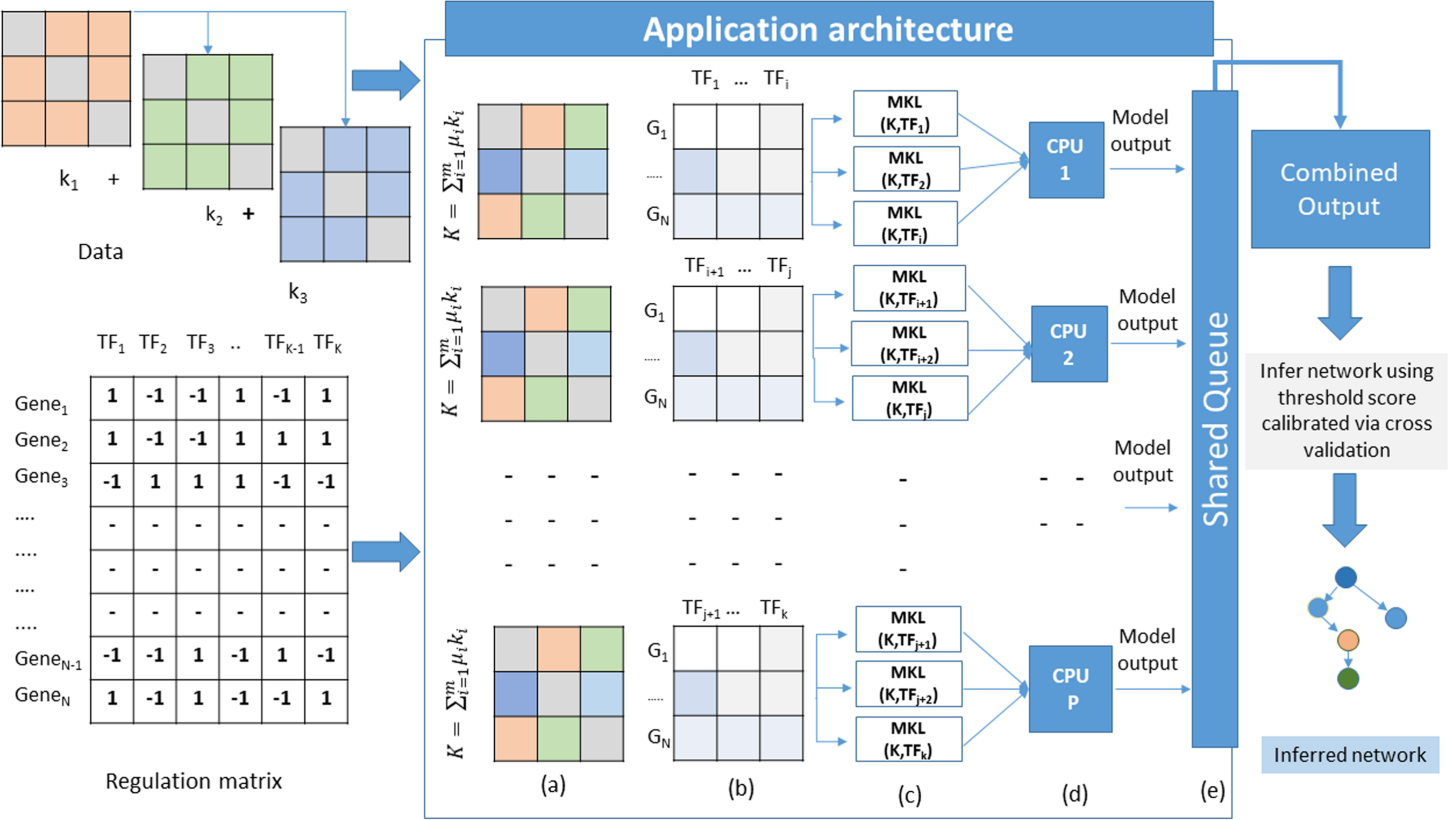
Application architecture of MKL-GRNI (A) Combined kernel (B) Decomposed regulation matrices (C) Parallel distribution and model building (D) Model execution (E) Writing results to shared object.

The proposed method adopts a supervised approach to learn new interactions between a TF and the whole genome of an organism. The algorithm operates on multiple datasets that characterize the genes of an organism. Since we are adopting an integrated approach, datasets such as gene expression, known TF-gene regulations, PPI, and DNA-methylation data can be combined using MKL approach. All these datasets are carefully chosen owing to their role in gene regulation. The TF-gene interaction data serves a dual purpose. It supplies the algorithm with prior knowledge about the regulatory relationships, and for each TF, the known target gene list also form the labels for the MKL classifier. For each TF, a set of known gene targets serve as positive examples. For negative examples, we divide our input into two subsets; the MKL classifier is trained using positive examples for which no prediction is needed, and the other subset contains negative examples. We perform 10-fold cross-validation using the same scheme and obtain discriminant values for all the genes with no prior regulation knowledge for this TF. This whole procedure is repeated for all the TFs. The idea here is to identify the set of genes whose expression profiles match those of positive examples even though the classifier is supplied with some false negative examples in the training set. A graphical overview of this architecture is depicted in Fig. 1. The problem of GRN inference from integrated datasets through supervised learning using MKL is not a trivial task. The nature of the complexity raises manifold while considering GRN inference of organisms with large genomes sizes. In this scenario, the model training and testing becomes TF specific. Therefore, the inference problem is decomposed into a set of classification subproblems corresponding to the total number of TFs present in the input Gene-TF interaction matrix. A sequential approach to such a problem scenario would require to run each subproblem one after the other in a loop. However, as we increase the number of TFs, the execution time of the algorithm also increases. To overcome such problems, we devise a strategy of parallel execution for the algorithm wherein multiple subproblems run simultaneously across different processors of a multi-processor hardware platform as explained in Algorithm 1.

**Algorithm 1:** MKL-GRNI Parallel approach for supervised inference of large-scale gene regulatory networks.

**Input:** *k* datasets *D*_1_, *D*_2_, . . . ., *D_k_*
**Input:** Regulation binary matrix *R* for Classification labels
**Output:** A matrix of decision scores *DS* for TF-Gene interaction
**begin**
*Transform D*_1_, *D*_2_, . . . ., *D_k_ int k*_1_, *k*_2_, . . . ., *k_n_ kernels using appropriate kernel function*
*Fuse n Kernels as K* = *k*_1_ + *k*_2_+…+*k_n_*
define mkl parameters *params* (*C*, *norm*, *epsilon*)
/* Distribute Source TF’s among multiple CPU’s*/
** foreach** *cpu in the cpu list* **do**
**do in parallel**
**foreach** *TF in source TF list* **do**
/* Set MKL parameters and Data */
set mkl.kernel ← *K*
set mkl.labels ←*R*
set mkl.parameters ← *params*
/* Obtain decision scores for MKL algorithm between each TF and all genes in the genomes*/
*DS_TF_ ← ApplyMKL*()
**end**
put *DS_TF_k__* in queue *Q*
**end**
**end**
**foreach** *q in Q* **do**
*DS_TF_k__ ← q.val*
**end**
**end**

Outputs generated by each model in the form of confidence scores (probability that a given TF regulates a gene) are stored in a shared queue object. Once all the subproblems finish their execution, the shared object is iterated to collect the results generated by all the models in order to build a single output matrix. In case the number of TFs is more than the number of available processors, they are split into multiple groups and dispatched to each processor with the condition that the number of TFs are divided in such a manner so that all the processors receive equal number of classification models.

### Kernel methods for genomic data fusion

**Figure 2:**
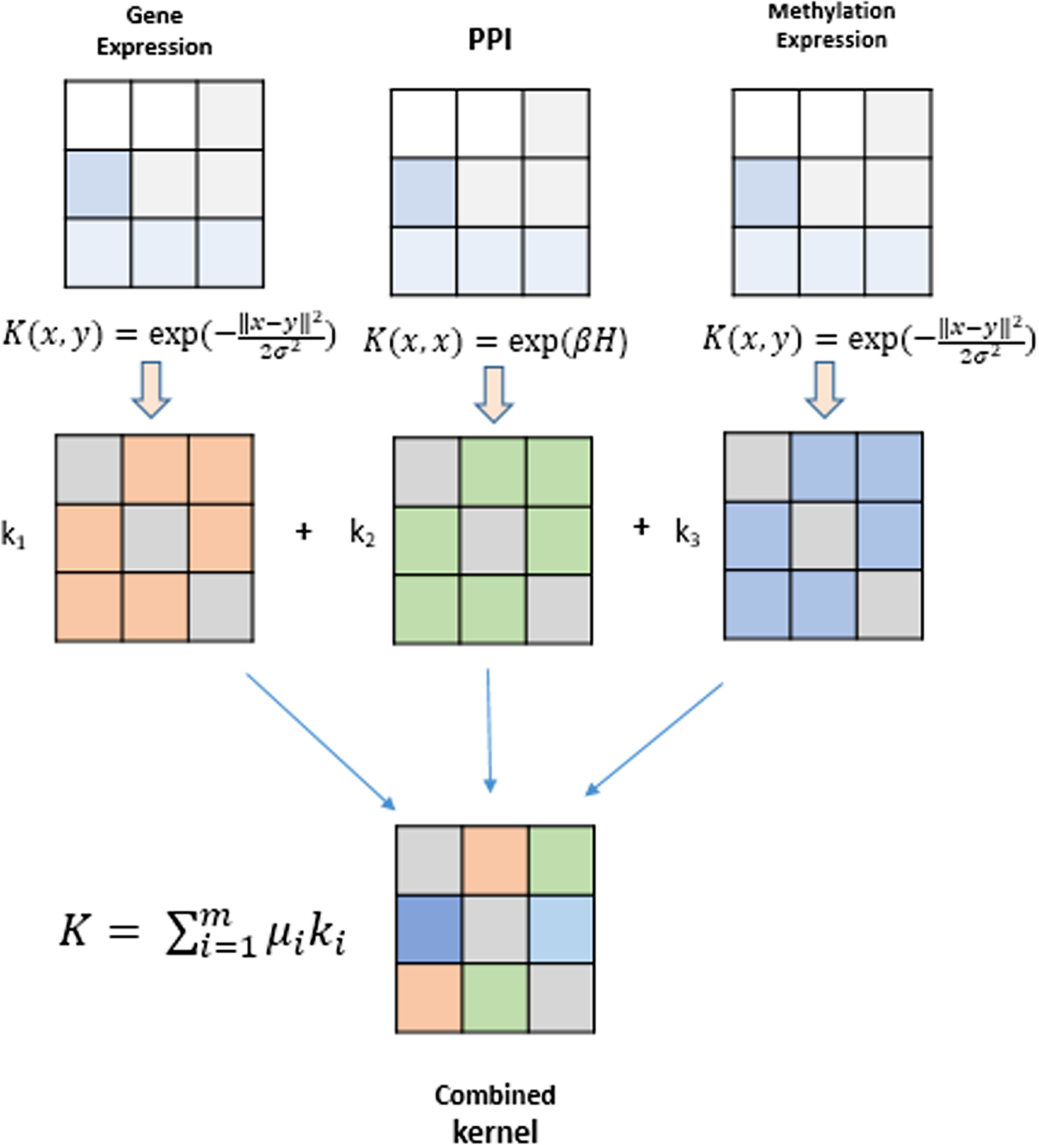
Genomic data fusion by combining kernel matrices from multiple kernels into a single combined kernel.

Kernel methods represent a mathematical framework which embeds data points (genes, proteins, drugs, etc) from input space*I*to feature space *F* by employing a kernel function. Genomic datasets viz., mRNA expression levels from RNA-seq, DNA methylation profiles and TF-gene regulation matrix obtained from different databases comprise heterogeneous datasets that can be fused using kernel methods and serve as the building blocks for inference of gene regulatory networks. A modular and generic approach to pattern analysis, kernel methods can operate on very high dimensional data in feature space by performing an inner product on the input data using a kernel function (Shawe-Taylor & Cristianini, 2004). An algorithm is devised that can work with such data and learn patterns. Such an algorithm is more generic as they operate on any data type that can be kernelized. These kernels are data specific, such as Gaussian, polynomial and sigmoid kernels for vectorial data, diffusion kernels for graph data, and string kernels for different types of sequence data. The kernel part is data specific, creating a flexible and modular approach to combine multiple modules to obtain complex learning systems. A graphical depiction of this fusion technique is shown in Fig. 2. The choice of different kernel functions for transforming datasets into their respective kernel matrices is made after a thorough analysis of literature in the field of kernel methods and MKL methods.

### MKL model

Multiple kernel learning is based on integrating many features of objects such as genes, proteins, drugs, etc., via their kernel matrices and represents a paradigm shift from machine learning models that use single object features (Sonnenburg et al., 2006). This combined information from multiple kernel matrices is provided as an input to MKL algorithm to perform classification/regression tasks on unseen data. Information represented by the kernel matrices can be combined by applying the basic algebraic operations, such as addition, multiplication, and exponentiation such that the positive semi-definiteness of the candidate kernels is preserved in the final kernel matrix. The resultant kernel can be defined by following equations using *k*_1_ and *k*_2_ as candidate kernel matrices and *ϕ*_1_(*x*) and *ϕ*_2_(*x*), their corresponding embedding in the feature space.

(1) $$K = {k_1} + {k_2}$$

with the new induced embedding

(2) $${\Phi _x} = {\Phi _1}(x),{\Phi _2}(x)$$

Given a kernel set *K* = {*k*_1_, *k*_2_, …, *k*_*m*_}, an affine combination of m parametrized kernels can be formed as given by: -

(3) $$K = \sum\limits_{i = 1}^m {\mu _i}{k_i}$$

subject to the constraint that *μ*_*i*_ (weights) are positive that is, μ_i_ ≥ 0, *i* = 1……..*m*. With these kernel matrices as input, a statistical classifier such as SVM separates the two classes using a linear discriminant by inducing a margin in the feature space. To find this discriminant, an optimization problem, known as a quadratic program (QP) needs to be solved. QP belongs to a class of convex optimization problems, which are easily solvable. Shogun toolbox solves this MKL optimization problem using semidefinite programing (SDP) first implemented for MKL learning by Lanckriet et al. (2004). Based on this margin, we classify SVM algorithms into hard, 1-norm soft and 2-norm soft margin SVM. Here we use the 1-norm soft margin SVM and SDP for MKL optimization and classification from heterogeneous datasets explained in our earlier work on MKL for biomedical image analysis (Wani & Raza, 2018). A detailed literature on SVM algorithms is covered in (Scholkopf & Smola, 2001).

### Datasets

To test the parallel MKL algorithm on multiple datasets, we downloaded gene expression data of *Escherichia coli* and *Saccharomyces cerevisiae* from DREAM5 Network inference challenge (Marbach et al., 2012) along with their gold standard network and human breast cancer transcriptomic data from TCGA. Some prominent features of these data are shown in Table 1.

**Table 1:** Dataset description of different organisms for supervised GRN inference.

Organism	Genes	Samples	Transcription factors	Known regulations	Known targets
*E. coli*	4,297	805	140	1,979	953
*S. cerevisiae*	5,657	536	120	4,000	2,721
Homo sapiens	19,201	1,212	66	73,052	12,028

Because the MKL paradigm provides the platform to fuse heterogeneous datasets, we download PPI data for both *E. coli* and *S. cerevisiae* from STRING database (Szklarczyk et al., 2011). The PPI data is supplied as prior biological knowledge to the algorithm in order to improve its inference accuracy as MKL can learn from multiple datasets. To supplement the human transcriptome with additional biological knowledge, we download DNA methylation expression data for all the genes in the transcriptome from the TCGA broad institute data portal (https://gdac.broadinstitute.org/). The regulation data (i.e., known interaction between genes and TFs) for *E.coli* and *S. cerevisiae* were extracted from the gold standard network provided in the DREAM dataset, however, for GRN inference in humans, the regulation data has been collected from a number of databases that store TF-gene interaction data derived from ChIP-seq and ChIP-ChIP experiments. We collected a list of 66 TFs from the ENCODE data portal (https://www.encodeproject.org/) for which ChIP-seq experiments were carried out on MCF7 breast cancer cell lines across different experimental labs. The targets of these TFs were extracted from ENCODE (ENCODE Project Consortium, 2004), TRED (Jiang et al., 2007) and TRRUST (Han et al., 2015) databases.

### Hardware and software requirements

The hardware platform used in this study is an IBM System X3650 M4 server model that includes an Intel Xeon processor having 24 cores and a primary memory of 32 GB with extendable option of 64 GB. The system supports a 64-bit memory addressing scheme having powerful 3.2 GHz/1066 MHz Intel Xeon processors with 1066 MHz front-side bus (FSB) and 4 MB L2 cache (each processor is dual core and comes with 2 × 2 MB (4 MB) L2 cache). The system also supports hyper threading features for more efficient program execution. In order to exploit this multi-core and multithreading features present in the hardware system we used multiprocessing Python package to dispatch different sub-problems across multiple cores of the computing system. The process of distribution of different learning sub-problems among different cores of a multi-core machine has been demonstrated in Fig. 1. For fusion of multiple datasets we use MKL approach whereby different datasets are first converted into similarity matrices (Kernels) and then joined to generate a final integrated matrix for learning TF-gene targets. We use MKL Python library provided by Shogun Machine Learning toolbox for implementing the proposed algorithm.

## Results

All the genomic datasets are transformed into their respective kernel matrices by using an appropriate kernel function. For example, datasets such as gene expression and DNA methylation expression data are transformed using a Gaussian radial basis function. The PPI data is converted into a diffusion kernel, *K* = *e*^βH^, where *H* is the negative Laplacian derived from adjacency and Degree matrix *H* = *A* − *D* of PPI graph. The TF-Target gene regulation data is organized as a binary matrix of labels (i.e., 1 and −1) with genes in rows and TFs in columns. The number of rows correspond to the genome size of the organism and the number of columns correspond to the total number of TFs being used for GRN inference. The elements of each column with value 1 signify that a gene *g*_i_ is regulated by TF_j_ and −1 otherwise. Such an organization of the regulation data allows us to use each column of the matrix as a label for individual classification problems in a supervised learning environment.

We perform two sets of experiments with our proposed approach in order to evaluate the scalability and the inference potential of the supervised learning from heterogeneous datasets using MKL paradigm. Our first experiment records execution times required to learn from varying genome and sample sizes on single and multi-processor architectures, given a set of TFs. Our second experiment focuses on the evaluation of inference potential of this approach on different genome and sample sizes. Since our problem of GRN inference is complex, the experiment aims to evaluate the parallel nature of the MKL algorithm by decomposing supervised inference of GRNs for multiple TFs into a number of subproblems and distribute them to multiple processors for parallel execution. Varying the genome and sample sizes in these experiments is to evaluate how efficiently MKL based models scale to large genomes where most of the GRN models developed till date do not perform optimally as reported in Marbach et al. (2012). The proposed method is implemented in Python and the code along with data is available at (https://github.com/waninisar/MKL-GRNI).

**Figure 3:**
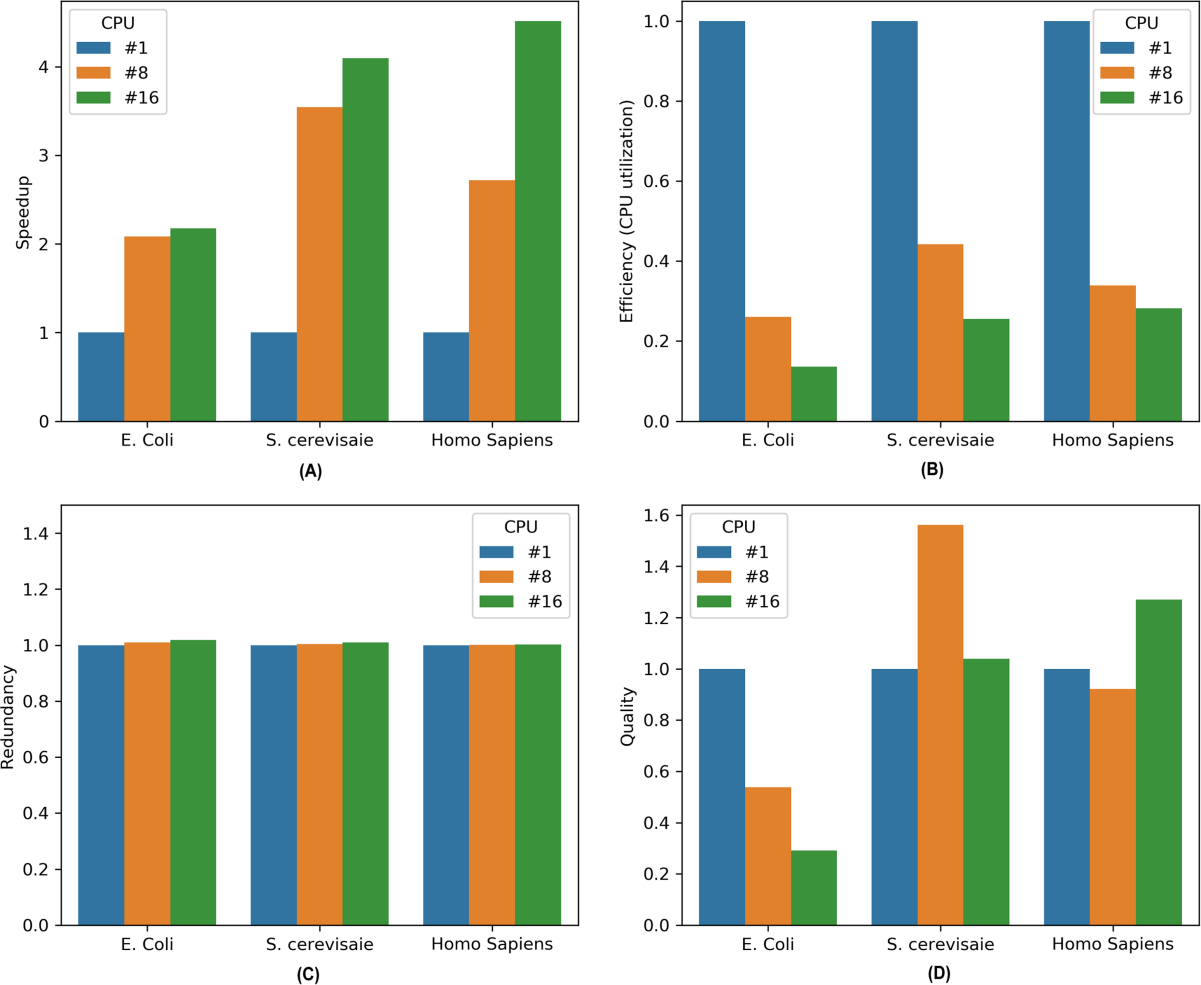
Performance metrics for parallel MKL-GRNI algorithm: (A) Speedup, (B) Efficiency, (C) Redundancy, (D) Quality.

To assess the performance of the parallel MKL-GRNI on different genomes characterized by datasets in Table 1. We execute the algorithm and embed the required code for the evaluation metrics. Once the algorithm completes its execution run, all the essential metrics are recorded for further analysis. The metrics are computed to evaluate the capacity of our approach in terms of reduced computational cost and enhanced inference accuracy when dealing with complex and large-scale inference tasks. Initially the algorithm is run in sequential mode for all the organisms for a set of 32 TFs, and later on in parallel mode on 8 and 16 CPUs. Performance metrics for all the datasets are plotted in Fig. 3. A brief description of these important performance metrics is given below:

## Speedup

We calculate speedup as a measure of relative performance of executing our algorithm in sequential and parallel processing environments. The speed up is calculated as under:-

(4) $$S(j) = T(1)/T(j)$$

Where *S*(*j*) is the speedup on *j* processors, *T*(1) is the time it takes on a single processor and *T*(*j*) is the time program takes on j processors.

## Efficiency

Efficiency is defined as the ratio of speedup to the number of processing elements (*j* CPUs in our case). It measures the utilization of the computation resources for a fraction of time. Ideally in parallel system, speedup is equal to *j* and efficiency is equal to 1. However, in practice, speedup is less than *j* and efficiency is between zero and one, depending on the effectiveness with which the processing elements are utilized. We calculate efficiency *E*(*j*) on *j* processors as given below:

(5) $$E(j) = S(j)/j$$

## Redundancy

Redundancy is computed as the ratio between number of operations executed in parallel and sequential modes. It measures the required increase in the number of computations when the algorithm is run on multiple processors.

(6) $$R(j) = O(j)/O(1)$$

## Quality

Quality measures the relevance of using parallel computation and is defined as the ratio between the product of speedup and efficiency to that of redundancy.

(7) $$Q(j) = S(j)xE(j)/R(j)$$

It is evident from the Fig. 1 that there is marked increase in the speedup as we move from a sequential (single CPU) to parallel execution (i.e., 8 and 16 CPUs). For an *E. coli* genome with a sample size of 500 and 32 TFs used for inference, the algorithm shows a sharp speedup as we move from sequential execution to parallel execution on 8 processors, however when the number of processors is increased to 16, there is marginal increase in speedup for *E. coli*. On the other hand, there is considerable increase in speedup recorded for 8 and 16 processors on higher genomes, such as *S. cerevisiae* and *Homo sapiens*, suggesting an increase in the capacity of the parallel algorithm to reduce the execution times. To assess the resource utilization using our parallel approach, the efficiency metric shows considerable drop in utilization of compute resources for all the three datasets, because only a section of algorithm runs in parallel. This can be inferred from the computed redundancy for sequential and parallel executions. The redundancy plot shows slight increase in terms of the computational cost incurred when running our computational problem in parallel, thereby suggesting less computational overhead as we switch from sequential to parallel mode of execution. To evaluate the relevance of parallel execution to our problem, we calculate quality metric for all the three datasets. From the barplots we can observe that parallel algorithms are less relevant when applied to smaller genomes as is evident in case of *E. coli*. But there is steady improvement in quality metric as move from *S. cerevisiae* to *Homo sapiens* with relevance indicator high when yeast dataset is run on 8 processors and human dataset on 16 processors. These improvements in speedup and quality metrics when running the algorithm in parallel provides us with a framework to work with more complex datasets and organisms with large genome sizes to infer very large scale GRNs using a supervised approach.

To assess the inference potential of this supervised method we compare the proposed approach with other methods that infer gene interactions from single and integrated datasets. Initially we apply MKL-GRNI to DREAM5 *E.coli* data, we performed a 10-fold cross-validation to make sure that model is trained on all the known regulations. At each cross-validation step, important performance metrics such as precision, recall and *F*1 score are recorded and then averaged for the whole cross-validation procedure. We then compared our network inference method with inference methods that predict TF-target gene regulations, such as CLR (Faith et al., 2007) and SIRENE (Mordelet & Vert, 2008). The results are recorded in Table 2.

**Table 2:** Average precision, recall and *F*1 measures for various inference methods.

Method	Average precision	Average recall	Average *F*1 score
CLR	0.275	0.55	0.36
SIRENE	0.445	0.73	0.55
MKL-GRNI	0.46	0.97	0.62

After running all the inference procedures, it is observed that the average precision , recall and F1 metrics generated by running MKL-GRNI is quite higher than those generated by other comparable methods. The improvement with MKL-GRNI can be attributed to the additional biological knowledge in the form of protein-protein interactions between *E.coli* genes to aid in the inference process.

To test the proposed method on integrated data, We perform a 10 fold cross-validation procedure on the input data. In this experiment, the known target genes of each organism as depicted in Table 1 are split into training and test sets. The model is trained on the features from the training set, and the network inference is performed between the genes in the test set, important evaluation metrics, such as Precision, Recall and *F*1 scores are recorded for each iteration and averaged across cross-validation runs. Table 3 summarizes these metric for varying genome and sample size for human breast cancer dataset and Table 4 contains results for all the three genomes.

**Table 3:** Precision, recall and *F*1 measure recorded for different combination of genome and sample sizes for Breast cancer data.

No. of genes	No. of samples	Average recall	Average Precision	Average F1 measure
5,000	100	0.8005	0.5817	0.6582
5,000	500	0.8005	0.6169	0.6848
5,000	1,000	0.8354	0.6347	0.6968
10,000	100	0.7350	0.4406	0.5509
10,000	500	0.7660	0.4537	0.5699
10,000	1,000	0.7860	0.4937	0.6065
19,201	100	0.7499	0.3746	0.4996
19,201	500	0.7444	0.3893	0.5112
19,201	1,000	0.7499	0.4246	0.5422

**Table 4:** Precision, recall and *F*1 measures averaged across cross-validation runs for complete genomes.

Organism	No. of genes	No. of samples	Avg. precision	Avg. recall	Avg. *F*1 measure
*E. coli*	4,297	802	0.46	0.97	0.62
*S. cerevisiae*	5,657	536	0.42	0.84	0.56
Homo sapiens	19,201	1,012	0.37	0.73	0.49

It is evident from these results that the MKL-GRNI algorithm scales well for higher genomes sizes. These metrics highlight the learning and inference potential of MKL. Looking at Table 3 we observe an average recall of 80% and an average precision of 58% with an average F1 measure of 65% for a genome size of 5,000 and sample size of 100, with an increase in these metrics as we increase the sample size to 500 and 1,000 respectively. However, as we start increasing the size of the genome, these metrics start a gradual decline for smaller sample size and again show a marginal increase as we increase the sample size for a fixed genome size. Although there is no direct rule of determining the number of samples corresponding to the size of the genome in omics studies, the improvements in precision, recall and *F*1 measures suggests an improvement in learning and inference potential of MKL algorithm with an increase in the number of samples. Also the tabulated metrics for all the three genomes in Table 4 show a considerable decline in the evaluation metrics as we move from smaller to larger genomes, suggesting a decrease in inference potential of the algorithm for larger datasets. The possible decline in the performance metrics can be attributed to increase in the genome size as we move from simple prokaryotic to more complex eukaryotic genomes. This increase in the genome sizes versus the sample size leads to curse of dimensionality and therefore making difficult to learn properly from skewed datasets.

We also compare our MKL-GRNI with a recently developed Integrative random forest for gene regulatory network inference (iRafNet) (Petralia et al., 2015). We select DREAM5 datasets of *E. coli* and *S. cerevisiae* and integrate PPI and gene expression data from both datasets. For MKL we build Gaussian and diffusion kernels from expression and PPI data. For iRafNet , the expression data serves as the main data and the PPI data is used as support data. Sampling weights are then derived from PPI data by building a diffusion kernel as *K* = *e*^H^ where *H* is a graph laplacian for PPI data. Sampling weights from *K* are derived as *W*^PPI^_i,__j_ = *K*(*i*, *j*) that is, the element *K* (*i*,*j*). The sampling weights thus obtained are then integrated with main data set (i.e., gene expression data). Putative regulatory links are then predicted using importance scores generated using the iRafNet R package. The AUC and AUPR scores obtained using iRafNet and MKL-GRNI are listed in Table 5.

**Table 5:** AUC and AUPR scores for *E. coli* and *S. cerevisiae* using iRafNet and MKL-GRNI.

Datasets	iRafNet		MKL-GRNI	
	AUC	AUPR	AUC	AUPR
*E. coli*	0.901	0.552	0.925	0.44
*S. cerevisiae*	0.833	0.39	0.89	0.42

The AUC and AUPR scores of MKL-GRNI thus obtained are comparable to iRafNet for both datasets. However, iRafNet reports a lower AUC and higher AUPR scores compared to MKL-GRNI when run on *E. coli* data. But once we move towards a higher genome size, these scores start dropping marginally for both iRafNet and MKL-GRNI approaches. The slight higher AUC scores in case of MKL-GRNI can be attributed to some extent to the skewed class label distribution where in negative labels far outnumber the positive ones because of limited known regulations. This class imbalance leads to higher predictive accuracy (AUC) but lower precision-recall scores (AUPR). On the other hand regression based GRN inference techniques have been reported to perform well for smaller genomes with GENIE3 (Huynh-Thu et al., 2010) being a start performer in DREAM5 network inference challenges. The higher AUPR generated by iRafNet in case of *E. coli* can be attributed to the way potential regulators are sampled using prior information from sampling weights (PPI), therefore decreasing false positives and increasing precision and recall. But for higher genomes (i.e, yeast in our case) the performance of both approaches begins to fall as reported by (Mordelet & Vert, 2008). Present implementation of iRafNet does not provide the ability to run the random forest algorithm in parallel. Therefore, using iRafNet for GRNI of higher genomes can incur huge computational cost by running thousands of decision trees in sequential mode. Since our main motive in this study is to parallelize the inference algorithm for large-scale GRNI, the higher speedup and higher quality provided by running MLK-GRNI in parallel can be used as a trade-off for slightly lower AUPR compared to iRafNet run in sequential mode with marginally higher AUPR scores.

## Discussion and Conclusion

Here we present a scalable and parallel approach to GRN inference using MKL as integration and supervised learning framework. The algorithm has been implemented in Python using Python interface to MKL provided by shogun machine learning toolbox (Sonnenburg et al., 2010). The ability of kernel methods in pattern discovery and learning from genomic data fusion of multi-omics data using MKL has already been demonstrated in a number of inference studies. Our focus here is to explore the scalability option for large-scale GRN inference in a supervised machine learning setting, besides assessing the inference potential across different genomes.

The approach undertaken can be considered as a parallel extension to SIRENE (Mordelet & Vert, 2008). Although SIRENE performs better than other unsupervised and information theoretic based inference methods as reported by (Mordelet & Vert, 2008). However, it lacks the ability to learn from heterogeneous genomic datasets that can provide essential and complementary information for GRN inference. Another limitation is the sequential execution of the TF-specific classification problems that incur the huge cost in terms of execution times as we move from *E. coli* genomes to more complex and large genomes of mice and humans. Therefore to facilitate very large scale GRN inference using supervised learning approach, we use the concept of decomposing the initial problems of learning GRN into many subproblems, where each subproblem is aimed to infer a GRN for a specific TF. Our algorithm distributes all such learning problems to different processors on a multi-processor hardware platform and dispatches them for simultaneous execution, thereby reducing the execution time of the inference process substantially. The results from each execution are written to a shared queue object, once all the child processes complete their execution, the queue object is iterated to build a single output matrix for genome-scale GRN inference. We also assess the inference potential of our MKL based parallel GRN inference approach by computing essential evaluation metrics for machine learning based approaches. A quick survey of scientific literature on GRN inference methods will ensure that the results obtained by our approach are comparable to other state-of-the-art methods in this domain and some cases better than inference methods that employ only gene expression data (e.g., CLR, ARACNE, SIRENE, etc. ). A drawback of our approach is that only TFs with known targets can be used to train the inference model. Also, the performance of the algorithm tends to decrease if the model training is carried out using TFs with few known targets, leading to a bias in favor of TFs with many known neighbors (i.e., hubs) and is less likely to predict new associations for TFs with very few neighbors. Besides, we are not able to identify new TFs among the newly learned interaction, nor the model can predict whether a given gene is upregulated or downregulated by a particular TF.

Therefore additional work is needed to improve the efficiency of the parallel algorithm and the inference potential of the MKL-GRNI. In our current implementation, we integrate only two datasets for GRNI, therefore leaving the scope to use more omics sources that can be integrated for improved performance of the inference model. Also, the MKL framework provides a mechanism to weigh the contribution of individual datasets that can be used to select informative datasets for integration. Further, we do not identify TFs from the predicted target genes and can be considered in future extension to this work. Besides, novel techniques to choose negative examples for training our parallel MKL-GRNI model can be incorporated to decrease the number of false positives and improve the overall precision/recall scores for genomes of higher organisms.

## Additional Information and Declarations

### Data Availability

The following information was supplied regarding data availability: The code is available at GitHub: https://github.com/waninisar/MKL-GRNI.

## References

[ref-1] Albert R (2007). Network inference, analysis, and modeling in systems biology. Plant Cell.

[ref-2] Alioscha-Perez M, Oveneke MC, Sahli H (2019). Svrg-mkl: a fast and scalable multiple kernel learning solution for features combination in multi-class classification problems. IEEE Transactions on Neural Networks and Learning Systems.

[ref-3] Ben-Hur A, Noble WS (2005). Kernel methods for predicting protein-protein interactions. Bioinformatics.

[ref-4] Butte AJ, Kohane IS (1999). Mutual information relevance networks: functional genomic clustering using pairwise entropy measurements. Biocomputing 2000.

[ref-5] Chen Z-Y, Fan Z-P (2014). Parallel multiple kernel learning: a hybrid alternating direction method of multipliers. Knowledge and Information Systems.

[ref-6] Cichonska A, Pahikkala T, Szedmak S, Julkunen H, Airola A, Heinonen M, Aittokallio T, Rousu J (2018). Learning with multiple pairwise kernels for drug bioactivity prediction. Bioinformatics.

[ref-7] ENCODE Project Consortium (2004). The ENCODE (ENCyclopedia Of DNA Elements) Project. Science.

[ref-8] Faith JJ, Hayete B, Thaden JT, Mogno I, Wierzbowski J, Cottarel G, Kasif S, Collins JJ, Gardner TS (2007). Large-scale mapping and validation of escherichia coli transcriptional regulation from a compendium of expression profiles. PLOS Biology.

[ref-9] Han H, Shim H, Shin D, Shim JE, Ko Y, Shin J, Kim H, Cho A, Kim E, Lee T, Kim H, Kim K, Yang S, Bae D, Yun A, Kim S, Kim CY, Cho HJ, Kang B, Shin S, Lee I (2015). TRRUST: a reference database of human transcriptional regulatory interactions. Scientific Reports.

[ref-10] Hecker M, Lambeck S, Toepfer S, Van Someren E, Guthke R (2009). Gene regulatory network inference: data integration in dynamic models: a review. Biosystems.

[ref-11] Huynh-Thu VA, Irrthum A, Wehenkel L, Geurts P (2010). Inferring regulatory networks from expression data using tree-based methods. PLOS ONE.

[ref-12] Jiang C, Xuan Z, Zhao F, Zhang MQ (2007). Tred: a transcriptional regulatory element database, new entries and other development. Nucleic Acids Research.

[ref-13] Kondor RI, Lafferty J (2002). Diffusion kernels on graphs and other discrete structures. Proceedings of the 19th International Conference on Machine Learning.

[ref-14] Lanckriet GR, De Bie T, Cristianini N, Jordan MI, Noble WS (2003). Kernel-based data fusion and its application to protein function prediction in yeast. Biocomputing 2004.

[ref-15] Lanckriet GR, De Bie T, Cristianini N, Jordan MI, Noble WS (2004). A statistical framework for genomic data fusion. Bioinformatics.

[ref-16] Lee TI, Rinaldi NJ, Robert F, Odom DT, Bar-Joseph Z, Gerber GK, Hannett NM, Harbison CT, Thompson CM, Simon I, Zeitlinger J, Jennings EG, Murray HL, Gordon DB, Ren B, Wyrick JJ, Tagne J-B, Volkert TL, Fraenkel E, Gifford DK, Young RA (2002). Transcriptional regulatory networks in saccharomyces cerevisiae. Science.

[ref-17] Marbach D, Costello JC, Küffner R, Vega NM, Prill RJ, Camacho DM, Allison KR, Consortium D, Kellis M, Collins JJ, Stolovitzky G (2012). Wisdom of crowds for robust gene network inference. Nature Methods.

[ref-18] Margolin AA, Nemenman I, Basso K, Wiggins C, Stolovitzky G, Dalla Favera R, Califano A (2006). Aracne: an algorithm for the reconstruction of gene regulatory networks in a mammalian cellular context. BMC Bioinformatics.

[ref-19] Mordelet F, Vert J-P (2008). SIRENE: supervised inference of regulatory networks. Bioinformatics.

[ref-20] Nascimento AC, Prudêncio RB, Costa IG (2016). A multiple kernel learning algorithm for drug-target interaction prediction. BMC Bioinformatics.

[ref-21] Pavlidis P, Weston J, Cai J, Noble WS (2002). Learning gene functional classifications from multiple data types. Journal of Computational Biology.

[ref-22] Petralia F, Wang P, Yang J, Tu Z (2015). Integrative random forest for gene regulatory network inference. Bioinformatics.

[ref-23] Raza K, Alam M (2016). Recurrent neural network based hybrid model for reconstructing gene regulatory network. Computational Biology and Chemistry.

[ref-24] Remli MA, Mohamad MS, Deris S, Samah AA, Omatu S, Corchado JM (2019). Cooperative enhanced scatter search with opposition-based learning schemes for parameter estimation in high dimensional kinetic models of biological systems. Expert Systems with Applications.

[ref-25] Scholkopf B, Smola AJ (2001). Learning with kernels: support vector machines, regularization, optimization, and beyond.

[ref-26] Seoane JA, Day IN, Gaunt TR, Campbell C (2013). A pathway-based data integration framework for prediction of disease progression. Bioinformatics.

[ref-27] Shawe-Taylor J, Cristianini N (2004). Kernel methods for pattern analysis.

[ref-28] Sonnenburg S, Henschel S, Widmer C, Behr J, Zien A, Bona Fd, Binder A, Gehl C, Franc V (2010). The shogun machine learning toolbox. Journal of Machine Learning Research.

[ref-29] Sonnenburg S, Rätsch G, Schäfer C, Schölkopf B (2006). Large scale multiple kernel learning. Journal of Machine Learning Research.

[ref-30] Speicher NK, Pfeifer N (2015). Integrating different data types by regularized unsupervised multiple kernel learning with application to cancer subtype discovery. Bioinformatics.

[ref-31] Szklarczyk D, Franceschini A, Kuhn M, Simonovic M, Roth A, Minguez P, Doerks T, Stark M, Muller J, Bork P, Jensen LJ, Mering C (2011). The STRING database in 2011: functional interaction networks of proteins, globally integrated and scored. Nucleic Acids Research.

[ref-32] Tomczak K, Czerwińska P, Wiznerowicz M (2015). The cancer genome atlas (tcga): an immeasurable source of knowledge. Contemporary Oncology.

[ref-33] Van Laarhoven T, Nabuurs SB, Marchiori E (2011). Gaussian interaction profile kernels for predicting drug-target interaction. Bioinformatics.

[ref-34] Wani N, Raza K (2018). Multiple kernel-learning approach for medical image analysis. Soft Computing Based Medical Image Analysis.

[ref-35] Wani N, Raza K (2019a). Integrative approaches to reconstruct regulatory networks from multi-omics data: a review of state-of-the-art methods. Computational Biology and Chemistry.

[ref-36] Wani N, Raza K (2019b). iMTF-GRN: integrative matrix tri-factorization for inference of gene regulatory networks. IEEE Access.

[ref-37] Yamanishi Y, Vert J-P, Kanehisa M (2004). Protein network inference from multiple genomic data: a supervised approach. Bioinformatics.

[ref-38] Yan S, Xu D, Zhang B, Zhang H-J, Yang Q, Lin S (2007). Graph embedding and extensions: a general framework for dimensionality reduction. IEEE Transactions on Pattern Analysis and Machine Intelligence.

